# The provision of recipes and single-use herb/spice packets to increase egg and protein intake in community-dwelling older adults: a randomised controlled trial

**DOI:** 10.1017/S1368980020002712

**Published:** 2021-06

**Authors:** E van den Heuvel, JL Murphy, KM Appleton

**Affiliations:** 1 Research Centre for Behaviour Change, Department of Psychology, Faculty of Science and Technology, Bournemouth University, Poole BH12 5BB, UK; 2 Faculty of Health and Social Sciences, Bournemouth University, Bournemouth, UK

**Keywords:** Egg intake, Dietary protein intake, Older adults, Randomised controlled trial

## Abstract

**Objective:**

This study investigated the impact of recipe and single-use herb/spice packet provision on egg intake and protein intake in community-dwelling individuals aged over 55 years.

**Design:**

Using a randomised-controlled intervention design, 100 older adults were randomised to receive (*n* 53) or not receive (*n* 47) high-protein egg-based recipes and herb/spice packets through the post for 12 weeks, from June to December 2016. Egg intake, protein intake, adverse events, lean body mass and functional measures of lean body mass were measured at baseline, after the 12 weeks and after a further 12 weeks.

**Setting:**

Bournemouth, UK.

**Participants:**

Community-dwelling older adults.

**Results:**

Intention-to-treat data were analysed using regression, controlling for various demographic and lifestyle characteristics. Ninety-three individuals (intervention, *n* 50; control, *n* 43) completed assessments at all three time points. Egg intakes increased by end of intervention in both groups (mean: 4–5 eggs/month). After a further 12 weeks, higher egg intakes were sustained in the intervention group, while egg intakes in the control group returned to baseline levels (between-group difference: *β* = −0·124, *P* = 0·047). No differences were found in other measures (largest *β* = −0·106, *P* = 0·12).

**Conclusions:**

The provision of high-protein egg-based recipes and single-use herb/spice packets over 12 weeks increased egg intakes up to 12 weeks after end of intervention. Other factors may explain increased egg intakes during the intervention, but the sustained effects most plausibly result directly from recipe provision. Limited effects in other measures suggest that the recipes may have replaced as opposed to added to existing protein intakes.

Low protein intake in older adults has been related to adverse health consequences, including low muscle mass and strength, with resultant impacts on muscle function, falls and frailty, mobility, independence and well-being^(1–3)^, and increasing protein intake can improve these outcomes^(4–7)^. Furthermore, not only is total protein intake important for health benefits, but also protein intake per meal has been associated with improved health outcomes^(8)^, and a threshold of 25–30 g of high-quality protein per meal is thought to be required for optimal stimulation of muscle protein synthesis in older adults^(9,10)^. Protein intake over the day for most older adults, however, is generally skewed to the main meal (a hot meal for either lunch or dinner) and is typically low for breakfast and other non-main meals^(11,12)^.

Protein-specific under-nutrition can affect up to 77 % of the older population^(11,13)^, the majority of whom live in the community. While under-nutrition is often treated with oral nutritional supplements in care settings^(14–16)^, these are expensive and can be wasteful and unacceptable to those who are more able and do not consider themselves to be ill^(17–21)^. For individuals who are more able, a food-based approach is recommended^(22)^. Protein intakes thus should be increased by encouraging the consumption of foods that are naturally high in protein^(22)^ and encouraging the consumption of protein-rich foods at breakfast or for other non-main meals may be particularly valuable.

A number of studies have used a food-based approach to increase protein intakes in older adults^(4,23–26)^. The foods used in these studies however often include meat and so may not be appropriate for non-main meals. By comparison, eggs are also a nutrient-dense source of high-quality protein^(27)^ and are generally considered appropriate for breakfast and smaller meals^(28)^. Eggs also have a soft texture, are easy to cook, of low cost and have a long shelf-life^(28–30)^, and are considered to add taste and variety to the diet^(31,32)^. All of these factors can encourage consumption, and specifically the consumption of protein-rich foods in older adults^(25,26,33–36)^.

In many intervention studies, the foods are also typically provided, while for long-term health benefits, an intervention must be sustainable, ideally through likely implementation by the target individuals themselves^(37,38)^. Studies using cooking lessons and classes report that older adults can learn to cook new meals, to add taste and variety to the diet and change dietary intakes^(39,40)^. Among these studies, participants also indicated that the recipes provided were one of the key contributors to behaviour change^(40)^. It remains unknown however, as to whether similar effects could be obtained in older adults when providing recipes without cooking classes. The results of one study (in younger adults) suggest that recipes can have similar impacts on dietary profiles to taking part in cooking lessons^(41)^, some interventions involving recipes have demonstrated a specific value for recipe provision^(42,43)^, and the recipes provided on food packages, in magazines, in supermarkets or as part of television programmes have been found to affect food choice^(44)^. Recipe provision may thus offer a low-cost intervention of benefit on a population-wide basis.

## Aim

This study aimed to investigate the impact of recipe provision on egg intake and protein intake, in community-dwelling individuals aged 55 years and over. Using a randomised controlled trial design, recipes and single-use herb/spice packets were provided, or not provided, to community-dwelling older adults over a 12 week period. Recipes were intended to increase flavour and variety in the diet and focused on breakfast meals to encourage egg and protein intake at a meal that is often low in protein. Herb/spice packets to make one portion of each recipe were also provided, where applicable, to encourage use of recipes. All outcomes were assessed at baseline, at the end of 12 weeks and after a further 12 weeks. Our primary outcomes were egg intake, protein intake and adverse events; secondary outcomes were lean body mass and functional measures of lean body mass. We hypothesised that the intervention, compared with control, would result in increases in all dietary intake and functional outcomes at 12 weeks and 24 weeks, compared with baseline.

## Methods

### Participants

Older adults were considered for inclusion in the study if they were: 55 years and over; living in their own homes; not allergic to eggs; not knowingly experiencing hypercholesterolaemia or familial hypercholesterolaemia; not knowingly experiencing renal insufficiency; not having undergone chemotherapy or radiotherapy in the last 6 months; not having a pacemaker or defibrillator; were not experiencing any condition or receiving treatment that affected eating or sense of flavour; able to give consent and complete all questionnaire measures and able to come to Bournemouth University, UK, for all test sessions. Individuals aged 55 years and over were included to test the intervention in a population group that may not only benefit from the intervention at the time but also in the future as a result of improvements in healthy habits^(22)^. Other inclusion criteria ensured against adverse health consequences as a result of involvement in the study^(45–48)^, while also allowing inclusion of a broad range of our target population. Our intervention was ultimately intended for population-wide use; thus, inclusion of a wide range of older adults living in the community was important. Participants were recruited via contact lists of individuals who had volunteered in previous studies, and by flyers and posters in the local community. Participants were not informed of the full aim of the study, to avoid effects as a result of demand characteristics^(49)^, but were instead told that the study was on ‘habits and lifestyles in older adults’.

#### Sample size

Our study was powered to detect an increase in mean daily protein intakes of 12·7 g/d, the difference between daily protein intakes in UK adults over 65 years (69·8 g/d) as recorded in the National Diet and Nutrition Survey^(50)^ and recommended intakes of 75–90 g (3 × 25–30 g)^(9,10)^. Assuming an *α* of 5 % for a power of 0·8, and no change in the control group, our required sample size was forty-one participants per group. Allowing for a possible dropout of 20 %, we aimed to recruit fifty participants per study group.

#### Randomisation

All eligible volunteers were randomised into one of the two groups: intervention or control. Randomisation was stratified per 10 year age group (55–64, 65–74, 75–85 and 85+ years) and by earlier involvement in related research^(31)^, using blocks of eight participants, with the exception that couples were always allocated to the same group. Randomisation was undertaken by a researcher with no direct contact with participants (K.M.A.), using sequential covered allocation lists.

### Intervention

Participants randomised to the intervention group received a set of six recipes every 2 weeks to their home address, for 12 weeks. Each recipe required one or two eggs and provided 25–30 g of protein per meal. The thirty-six different recipes were gained from the website www.eggrecipes.co.uk, developed by the British Egg Industry Council^(51)^, were selected as suitable for breakfast/brunch and amended to contain 25–30 g of protein. Each set of recipes included a variety of egg preparations (e.g. fried eggs, scrambled eggs, omelettes); a variety of preparation times and methods; meat, fish and vegetarian dishes and a variety of traditional and foreign flavours. Recipes were printed in large clear text on glossy A5 recipe cards, to aid durability and simulate supermarket recipe cards, and included preparation time and nutritional composition, with the protein content highlighted in bold. All recipes mentioned the dish was high in protein and recommended consumption for breakfast or brunch. Recipe cards were pilot tested prior to the study with individuals in the target age group, and the text on several recipes was altered for face validity as a result. A list of all recipes can be found in online Supplementary Material I.

All the herbs/spices (in individual plastic pouches) which were required for one preparation of each dish were also provided. Herbs/spices were provided to enable use of the recipes without additional ingredient expense. Other ingredients were not provided to ensure that the intervention could be implemented in a real-life scenario.

Participants were not instructed to use the recipes, or given any further information regarding their intended use or impact. The intervention was intended to mirror the provision of recipes by a supermarket or magazine, where shoppers or readers would also receive no additional instruction. To increase the relevance of the recipes to older adults, all participants also received a dietary information postcard, detailing the importance and benefits of dietary protein for older adults, particularly when consumed for breakfast. The dietary information postcard mentioned eggs among other high-protein foods but made no reference to the recipes or other aspects of the study, and was identical for all participants (intervention and control groups).

### Control

Participants in the control group did not receive any recipes, herbs or spices, but received all other study documentation (study information sheet, inclusion/exclusion criteria, consent form, testing schedules, questionnaires and dietary information postcard) and undertook all test sessions in the same manner as the intervention group.

### Primary outcomes

Our primary outcomes were egg intake, protein intake and adverse events.

#### Egg intake

Egg intake was measured using an egg consumption FFQ^(32)^, and the Scottish Collaborative Group FFQ (SCG-FFQ)^(52,53)^. The egg consumption FFQ requested frequency of consumption of eighteen preparations of eggs: boiled eggs (hot), hard boiled eggs (cold), fried eggs, scrambled eggs, poached eggs, omelettes, scotch eggs, quiches/savoury flans, egg sandwiches, egg salad, egg mayonnaise, custards, meringues, sweet flan/crème caramel, duck/quail’s eggs, raw eggs, egg yolk separate from the white and egg white separate from the yolk. For each type of egg, participants were asked to complete number of measures (e.g. one egg, one slice) consumed per day (1, 2, 3, 4 and 5+) on number of days per week, using response options 1–7 d/week, once or twice per month or rarely/never. Responses were converted to number of eggs eaten per month. All foods were considered to contribute equal portions of eggs, excepting the dishes egg mayonnaise, custards, meringues and sweet flan/crème caramel. Responses for egg mayonnaise were discounted due to varying portion sizes and limited contribution to daily egg intakes, but egg mayonnaise was retained on the questionnaire to increase face validity. Responses for the sweet dishes were considered as 0·5 portions because a standard portion is commonly composed of less than one egg^(28)^.

The SCG-FFQ is a validated FFQ, developed by the Scottish Collaborative Group, measuring dietary intake over the last 2–3 months^(52,53)^. The section on egg intake consists of three questions: number of measures (eggs) per day, and the number of days per week that respondents consume ‘Boiled or poached eggs’; ‘Fried eggs’ and ‘Scrambled eggs or omelette’. Response options were used and converted to monthly egg intake as above.

#### Protein intake

Protein intake was measured using the complete SCG-FFQ, where food intake data were converted to protein intake and protein intake from animal sources, by the Scottish Collaborative Group^(52)^. Protein intake from animal sources consisted of protein intake from meat, fish, eggs and dairy foods. Animal-based proteins contain all essential amino acids, are considered high-quality protein sources^(54)^ and stimulate a higher net protein synthesis than plant protein sources^(55)^.

#### Adverse events

To assess adverse events, participants were asked whether they had experienced any of the following more or less often than usual, during the previous month: nausea, digestive issues (e.g. constipation or diarrhoea), stomach aches/cramps, hunger, bloating/uncomfortable fullness, thirst, headaches, fatigue/tiredness, restlessness, dizziness, skin rashes or any other experiences that were unusual. Positive reports were queried for possible reasons.

### Secondary outcomes

Our secondary outcomes were lean body mass and functional measures of lean body mass: physical performance; leg strength and hand-grip strength. Lean body mass from fasting resting body composition was assessed by bioelectrical impedance analysis with the use of a 50-kHz generator (1500 MDD; Bodystat), validated for use in older adults. Physical performance was assessed using a short physical performance battery^(56)^, consisting of three physical functioning tests measuring: lower body strength (chair stands); standing balance and walking speed (8-foot walk (2·4 m)). Leg strength was measured in a seated position by counting the number of times participants could extend their leg wearing no weights, 2·5 or 5 kg ankle weights in a 30 or 60 s period^(57)^. Handgrip strength was measured using a handgrip dynamometer (Takei, GRIP-D, T.K.K. 5401)^(58)^. Participants were asked to undertake three measures of handgrip strength for each hand, alternating between right and left hands. The maximum of the six measures was used for analyses^(59)^.

### Additional outcomes

Variables (as below) which have also previously been associated with egg intake, protein intake, protein status or eating behaviour in older adults were also measured as potential confounders^(21,33,34,60–65)^.

#### Demographic and lifestyle characteristics

Demographic and lifestyle characteristics: date of birth; gender; marital status; living status; years of education; nationality; most recent level of employment; frequency of help with food shopping or preparation, of eating out or away from the home and of food delivery; vegetarianism, pescatarianism or veganism; and denture wearing; were assessed by a self-report questionnaire. Food neophobia, a measure of willingness to try new foods, and risk of sarcopenia were also assessed using validated questionnaires^(66,67)^.

#### Energy intake

Energy Intake was assessed as part of the SCG-FFQ by the Scottish Collaborative Group^(52)^, by consideration of all foods.

#### BMI

BMI was calculated from measured standing height and body weight in light clothing, without shoes (stadiometer (SECA gmbh & Co., accurate to 0·1 cm); calibrated scale (The Boots Company PLC, accurate to 0·1 kg)).

#### Usual physical activity

Energy expended in physical activity per week was assessed using the Community Healthy Activities Model Program for Seniors, a physical activity questionnaire for older adults^(68)^. The Community Healthy Activities Model Program for Seniors lists a variety of light, moderate and vigorous physical activities and requests weekly frequency of performing each activity in number of hours per week. Responses were converted to estimated energetic expenditure per week on all activities^(68)^.

#### Health-related quality of life

Health-related quality of life was assessed using the short-form thirty-six-item questionnaire (SF-36)^(69)^. Scores for all nine domains were generated^(69)^ and added up to give a total SF-36 score, where a higher score suggests a greater health-related quality of life.

#### Reasons for eating or not eating eggs

Reasons for eating/not eating eggs were assessed by the questionnaire previously developed^(32)^, to give insights into possible barriers to trying the recipes.

#### Recipe feedback

Intervention group participants also received a recipe feedback form after the second and third test sessions, asking for comments on which recipes and herb/spice packets were used or not used, and why.

### Procedure

**Fig. 1:**
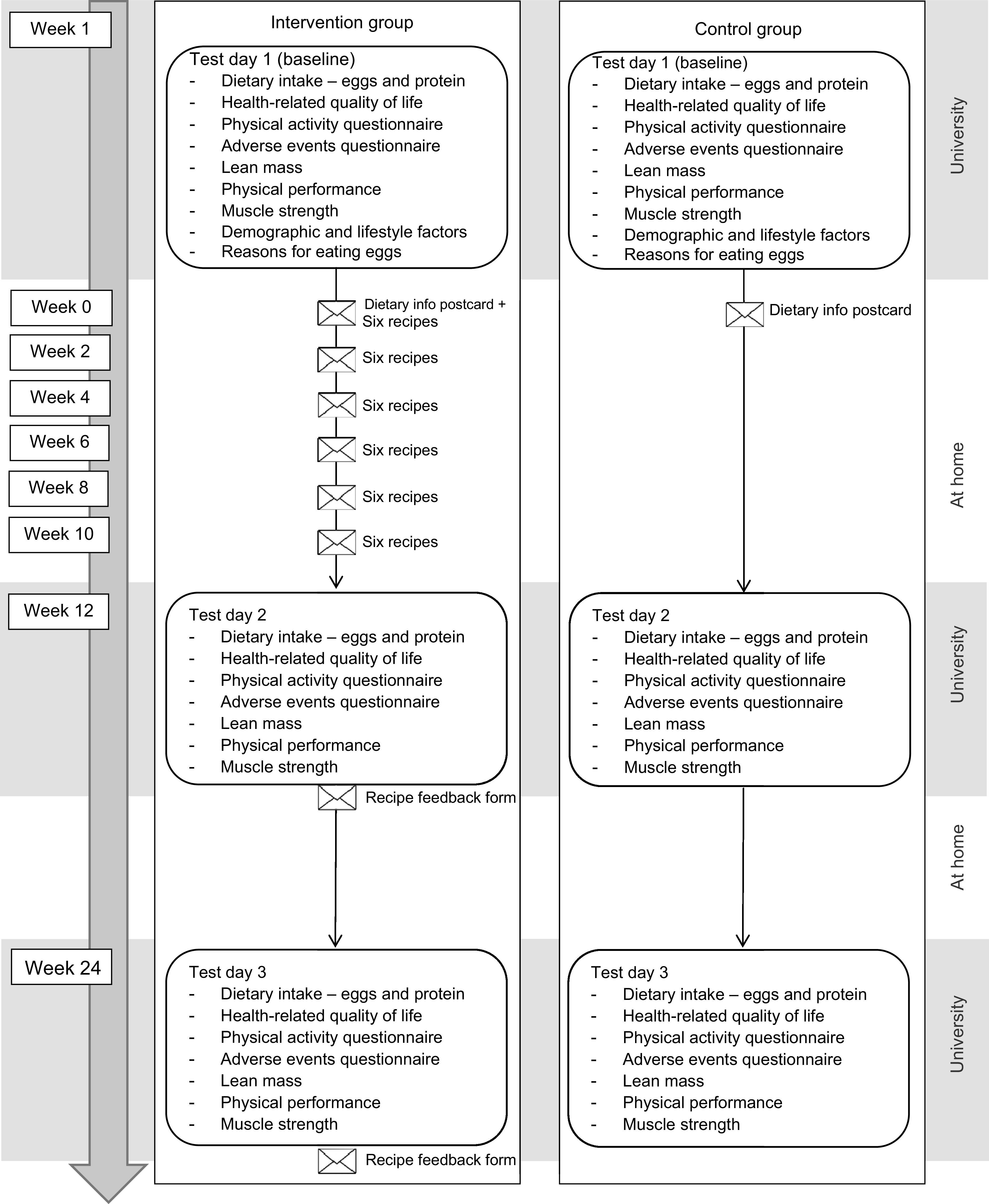
Schematic overview of the study

A schematic overview of the study design can be found in Fig. 1. All participants came to Bournemouth University for a test session at baseline (T1), after 12 weeks intervention (T2) and after a further 12 weeks (T3). All test sessions were conducted in the morning between 8.00 and 11.00 hours, at a time of the participant’s preference. A week before each session, participants received a set of questionnaires through the post to complete at a time suitable to them and bring with them. All questionnaires were completed in relation to the previous 4 weeks. Participants came to the test session fasted and were asked to have had only water to drink since going to sleep the night before. Height and weight were measured first, followed by bioelectrical impedance measures of body composition. After these measures, a breakfast of toast/cereal and coffee/tea was provided, during which all questionnaires were checked for missing values and any queries were discussed; then, the functional measures of lean muscle mass (physical performance and muscle strength) were undertaken. On a few occasions where several queries arose in relation to the questionnaires, these were addressed by the researcher and participants finished the questionnaires at home within a few days of the test session and returned them by post. Test sessions were individual, or if preferred per couple, and lasted approximately 1 h per person. Questionnaires and tests were the same for each test session (T1, T2 and T3), excepting that demographic and lifestyle characteristics, and reasons for eating/not eating eggs were assessed only at baseline. After the baseline test session, all participants also received a postcard with a short dietary information message, as above. The procedure was the same for all participants (intervention and control groups).

#### Blinding

Recipes and herb/spice packets were sent to intervention participants by a researcher with no direct contact with participants (K.M.A.). All outcome assessments were undertaken by a researcher who was blind to allocated condition for each participant (intervention/control) (E.v.d.H.), and participants in the intervention group were asked not to mention the recipes to the researcher during test sessions. Participants were not blind to condition (participants knew whether they were receiving recipes or not), although they were blind to other possible conditions in the study.

### Trial registration

The study was registered at ClinicalTrials.gov (NCT02777918) in May 2016. Test sessions were run from June 2016 to March 2017. All methods were undertaken as detailed in the trial registration.

### Data analyses

Data were analysed using IBM SPSS Statistics for Windows, version 22, using an Intention-to-treat approach. First, all measures were tested for normality. Demographic and lifestyle characteristics were used to describe the sample. All baseline measures were compared between the intervention and control groups using *χ* ^2^ tests and Mann–Whitney *U* tests, and correlations were investigated between all baseline measures. Multiple linear regression analyses were then used for the main analyses^(70)^. Multiple linear regression was used to allow investigation of outcome measures taking simultaneous account of baseline measures and a number of potentially confounding variables and was considered most suitable given our primary interest in final outcome and the number of potential confounders^(70)^. Alternative analyses, for example, ANOVA, would not have allowed for these considerations. The effect of the intervention on all outcome measures was assessed controlling for age, gender, baseline measures of the dependent variable, previous participation in related studies, and measures of protein intake, BMI, usual physical activity and HR-QoL score as assessed at the same time. Previous participation was included in regression models to allow for possible demand characteristics. Energy intake was not included in regression models following high correlations with BMI. The regression models also did not include other demographic and lifestyle characteristics to maintain statistical power^(71)^. Similarly, only a single score for some measures that can be divided to provide subscores, for example, HR-QoL, was used. Means and standard deviations for SF-36 subscales per group per time point are given in online Supplementary Material II. Separate regression models were used to predict all primary and secondary outcomes, at both the end of the 12 week intervention (T2) and at the end of the following 12 weeks (T3). Egg intakes were analysed primarily using the egg consumption FFQ and secondarily using the SCG-FFQ measure. Additional data, for example, on reasons for eating/not eating eggs and recipe feedback, were explored in the intervention group only and will be published elsewhere in exploratory analyses investigating intervention success/failure. Missing data were completed using last observation carried forward. There were no concerns over multi-colinearity between predictor variables. Data are reported using means and standard deviations. Significance was taken as *P* ≤ 0·05.

## Results

### Participants

A total of 100 participants took part in the study: fifty-four females and forty-six males with a mean age at baseline of 70 (sd 7) years, range 55–97 years. Following randomisation, fifty-three participants were allocated to the intervention group, forty-seven participants to the control group. There were no significant differences between groups excepting in frequency of food delivery (*χ* ^2^(2) = 16·36, *P* < 0·01), and sarcopenia prevalence based on the sarcopenia screening tool (*χ* ^2^(1) = 4·35, *P* < 0·05). Participant characteristics at baseline are given in Table 1.

**Table 1:** Participant characteristics*†

	Total sample (*N* 100)	Intervention group (*n* 53)	Control group (*n* 47)
Age (years)			
Mean	70	70	70
sd	7	8	7
Gender			
Female	54	30	24
Male	46	23	23
Education (years)			
Mean	15	15	15
sd	3	3	3
Marital status			
Married	66	33	33
Divorced	19	10	9
Widowed	8	6	2
Never married	7	4	3
Living status			
Alone	30	17	13
With others	70	36	34
Most recent employment level			
Unemployed	2	1	1
Manual worker	10	4	6
Non-manual worker	29	15	14
Professional/management	57	32	25
Vegetarian/pescatarian/vegan			
No	95	50	45
Yes	5	3	2
Denture wearing			
No	84	45	39
Partial dentures	13	5	8
Full dentures	3	3	0
Receiving help with food shopping			
Never	95	51	44
Sometimes	3	1	2
Often	1	1	0
Receiving help with food preparing			
Never	95	51	44
Sometimes	4	2	2
Often	0	0	0
Eating out or away from home			
Never	2	1	1
Sometimes	74	38	36
Often	23	13	10
Getting food delivered‡			
Never	85	**42**	**43**
Sometimes	13	**10**	**3**
Often	0	**0**	**0**
Food neophobia			
Mean	22	22	22
sd	7	7	7
Sarcopenia prevalence (screening tool score)§			
*n*	4	**3**	**1**
%	4	**6**	**2**

* Measures are reported as frequencies or mean and sd. Variables with significant differences between groups are in bold.

† For some variables, the sample is not complete because a few participants left a question open.

‡ Significant difference between the intervention and control groups (*P* < 0·01).

§ Significant difference between the intervention and control groups (*P* < 0·05).

### Adherence

**Fig. 2:**
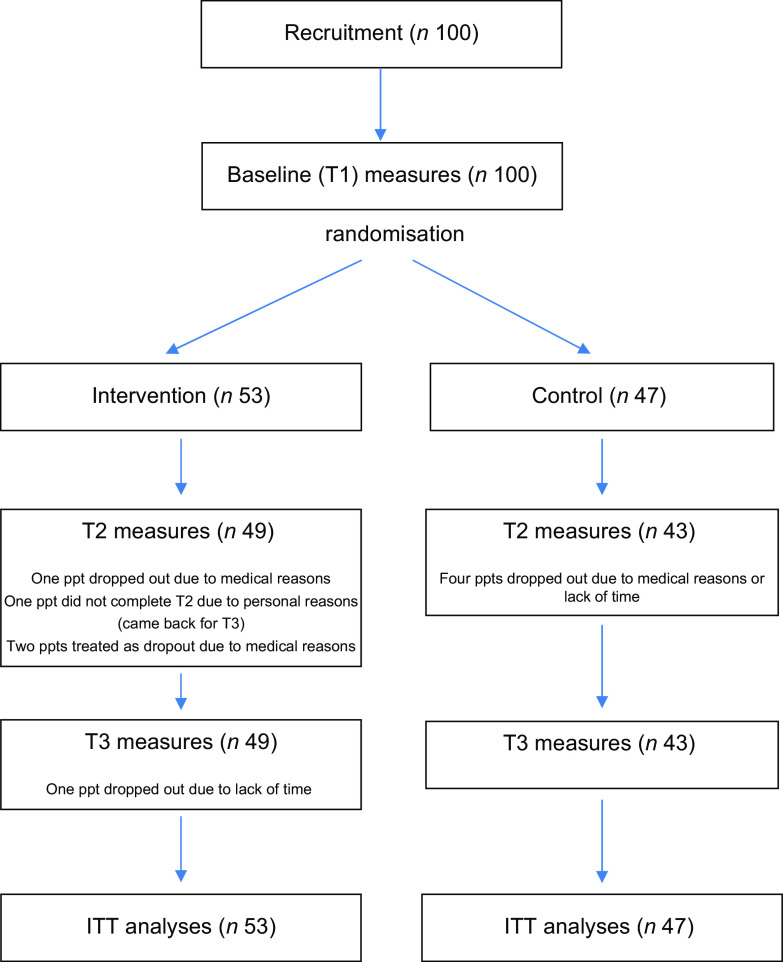
Schematic demonstrating the flow of participants through the study. ITT, intention-to-treat

A total of ninety-three participants completed all three sessions (fifty participants in the intervention group, forty-three participants in the control group). Five participants dropped out of the study after T1 (one participant in the intervention group, four participants in the control group), for reasons not related to the intervention (medical reasons (*n* 3) and time required for the sessions and questionnaires (*n* 2)). One participant (intervention group) dropped out after T2. One participant (intervention group) missed T2, but came back for the T3 session. Two additional participants (intervention group) completed all three test sessions, but suffered a medical condition affecting their diet and ability to change their diet in between test sessions. They were treated like drop outs after T1 for analyses. In addition, one individual completed all measures for all three test sessions but all questionnaire-based measures were excluded from data analyses for exceptionally high reported egg intake (about fifteen eggs/d). A schematic demonstrating the flow of participants through the study is given in Fig. 2.

Fifty-eight percent of the individuals in the intervention group reported using the recipes.

Blinding of the researcher undertaking assessments was broken by some participants during the trial (*n* 14). These incidences were recorded, and there were no differences in the measures taken by the researcher after the blinding was broken between the participants who broke the blinding and those who did not.

#### Baseline measures

All baseline measures for the total sample, and for the intervention and control group, can be found in Table 2. Mann–Whitney *U* tests revealed only one significant difference between groups: short physical performance battery score was lower for the intervention group than for the control group (*U* = 1692·5, *z* = 3·11, *P* < 0·01). Spearman correlations showed relationships between the baseline measures and demographic characteristics as would be expected.

**Table 2:** Baseline measures for all participants, intervention and control groups*

	Total sample (*N* 100)		Intervention group (*n* 53)		Control group (*n* 47)	
	Mean	sd	Mean	sd	Mean	sd
Egg intake (eggs/month)	22	16	23	17	21	15
Protein intake (total) (g/d)	92	32	93	30	91	33
Protein intake (total) (g/d per kg body weight)	1·26	0·46	1·25	0·43	1·27	0·49
Protein intake (animal sources) (g/d)	56	21	56	20	55	21
Protein intake (animal sources) (g/d per kg body weight)	0·76	0·29	0·75	0·27	0·76	0·31
Adverse events (0–19)	0·91	1·30	1·08	1·21	0·72	1·38
Lean body mass (g)	50·42	12·68	50·36	12·68	50·49	12·81
SPPB score (0–12)†	8·52	2·37	**7·81**	**2·34**	**9·32**	**2·16**
Handgrip strength (kg)	32·61	10·00	31·58	10·51	33·78	9·36
Leg extensions (extensions)‡	31	10	32	10	29	8
BMI (kg/m^2^)	26·64	4·34	26·98	4·18	26·26	4·53
Energy intake (kJ/d)	9155	3160	9188	2872	9121	3491
Physical activity§ (kJ/week)	18 075	12 734	17 577	13 069	18 653	12 458
HR QoL score** (0–900)	684	131	681	138	687	123

SPPB, short physical performance battery; HR QoL, health-related quality of life.

* All measures are reported as number (*n*) or mean and sd. Variables with significant differences between groups are in bold.

† Significant difference between the intervention and control groups (*P* < 0·01).

‡ Leg extensions are counted for different durations and using different ankle weights between participants. Mean values should be interpreted carefully.

§ Physical activity was measured by the Community Healthy Activities Model Program for Seniors questionnaire.

** HR QoL was measured by the SF-36 questionnaire.

### Primary outcomes

Means and standard deviations for all outcomes are given in Table 3. Results of all regression analyses for primary outcomes are given in Tables 4 (T2) and 5 (T3). All regression models were significant.

**Table 3:** Means and standard deviations for all outcome measures per group per time point*

	T1		T2		T3	
	Mean	sd	Mean	sd	Mean	sd
Intervention group (*n* 53)						
Egg intake (eggs/month)	23	17	28	20	27	22
Protein intake (total) (g/d)	93	30	90	29	86	36
Protein intake (total) (g/d per kg body weight)	1·25	0·43	1·21	0·43	1·14	0·48
Protein intake (animal sources) (g/d)	56	20	55	23	53	24
Protein intake (animal sources) (g/d per kg body weight)	0·75	0·27	0·74	0·31	0·70	0·31
Adverse effects (0–19)	1·08	1·21	1·15	1·86	1·36	1·46
Lean body mass (g)	50·36	12·68	50·02	12·62	49·99	12·58
SPPB score (0–12)	7·81	2·34	8·38	2·56	8·40	2·41
Handgrip strength (kg)	31·58	10·51	32·37	10·68	32·48	9·93
Leg extensions† (extensions)	32	10	31	11	32	11
BMI (kg/m^2^)	26·98	4·18	27·10	4·05	27·33	4·45
Energy intake (kJ/d)	9188	2872	8925	2738	8460	3265
Physical activity‡ (kJ/week)	17 577	13 069	14 333	10 905	16 334	10 929
HR QoL score§ (0–900)	681	138	698	134	696	136
Control group (*n* 47)						
Egg intake per month (eggs)	21	15	25	20	22	15
Protein intake (total) (g/d)	91	33	94	35	90	31
Protein intake (total) (g/d per kg body weight)	1·27	0·48	1·31	0·49	1·26	0·51
Protein intake (animal sources) (g/d)	55	21	59	25	54	24
Protein intake (animal sources) (g/d per kg body weight)	0·76	0·31	0·81	0·34	0·75	0·39
Adverse events (0–19)	0·72	1·38	1·06	1·26	1·19	1·71
Lean body mass (g)	50·49	12·81	50·21	12·76	50·12	12·72
SPPB score (0–12)	9·32	2·16	9·13	2·09	8·81	2·26
Handgrip strength (kg)	33·78	9·36	34·04	9·65	33·60	9·60
Leg extensions† (extensions)	29	8	29	10	30	10
BMI (kg/m^2^)	26·26	4·53	26·35	4·31	26·50	4·56
Energy intake (kJ/d)	9121	3491	8966	3102	8786	2738
Physical activity‡ (kJ/week)	18 649	12 458	14 944	9640	15 601	10 118
HR QoL score§ (0–900)	687	123	703	131	703	131

SPPB, short physical performance battery; HR QoL, health-related quality of life.

* Measures are reported as number (*n*) or mean and sd.

† Leg extensions are counted for different durations and using different ankle weights between participants. Mean values should be interpreted carefully.

‡ Physical activity was measured by the Community Healthy Activities Model Program for Seniors questionnaire.

§ HR QoL was measured by the SF-36 questionnaire.

**Table 4:** Multiple linear regression results predicting egg intake, protein intake and adverse events after the 12 week intervention (T2) (*n* 100)*

Regression model	Egg intake		Protein intake (total)		Protein intake (animal sources)		Adverse events	
	*R* = 0·816, *R* ^2^ = 0·666, adjusted *R* ^2^ = 0·632, *F*(9, 89) = 19·709, and *P* < 0·001		*R* = 0·778, *R* ^2^ = 0·605, adjusted *R* ^2^ = 0·570, *F*(8, 90) = 17·226, and *P* < 0·001		*R* = 0·713, *R* ^2^ = 0·508, adjusted *R* ^2^ = 0·464, *F*(8, 90) = 11·609, and *P* < 0·001		*R* = 0·600, *R* ^2^ = 0·360, adjusted *R* ^2^ = 0·295, *F*(9, 89) = 5·564, and *P* < 0·001	
	*β*	*P*	*β*	*P*	*β*	*P*	*β*	*P*
Condition (intervention, control)	−0·013	0·84	0·071	0·29	0·095	0·21	0·051	0·56
Age (years)	0·013	0·84	−0·031	0·67	−0·019	0·81	−0·097	0·29
Gender (female, male)	0·017	0·80	0·093	0·21	0·072	0·38	−0·125	0·19
Egg intake at T1 (eggs/month)	**0·685**	**<0·01**						
Protein intake (total) at T1 (g/d)			**0·749**	**<0·01**				
Protein intake (animal sources) at T1 (g/d)					**0·681**	**<0·01**		
Adverse events at T1							**0·409**	**<0·01**
Protein intake (total) at T2 (g/d)	**0·200**	**<0·01**					0·037	0·68
BMI atT2 (kg/m^2^)	**0·154**	**0·02**	−0·028	0·69	0·024	0·76	0·141	0·13
Physical activity at T2 (kJ)†	−0·012	0·86	−0·094	0·20	−0·067	0·41	0·062	0·51
HR QoL score at T2‡	−0·098	0·15	−0·088	0·22	−0·110	0·17	−0·192	0·05
Previous participant (no, yes)	−0·019	0·79	0·109	0·13	0·065	0·43	−0·043	0·65

HR QoL, health-related quality of life.

* Significant co-efficients are in bold.

† Physical activity was measured by the Community Healthy Activities Model Program for Seniors questionnaire.

‡ HR QoL was measured by the SF-36 questionnaire.

**Table 5:** Multiple linear regression results predicting egg intake, protein intake and adverse events after the 12 week follow-up (T3) (*n* 100)*

	Egg intake		Protein intake (total)		Protein intake (animal sources)		Adverse events	
	*R* = 0·820, *R* ^2^ = 0·673, adjusted *R* ^2^ = 0·640, *F*(9, 89) = 20·352, and *P* < 0·001		*R* = 0·720, *R* ^2^ = 0·519, adjusted *R* ^2^ = 0·476, *F*(8, 90) = 12·143, and *P* < 0·001		*R* = 0·730, *R* ^2^ = 0·532, adjusted *R* ^2^ = 0·491, *F*(8, 90) = 12·807, and *P* < 0·001		*R* = 0·603, *R* ^2^ = 0·364, adjusted *R* ^2^ = 0·299, *F*(9, 89) = 5·650, and *P* < 0·001	
Regression model	*β*	*P*	*β*	*P*	*β*	*P*	*β*	*P*
Condition (intervention, control)	**−0**·**124**	**0**·**047**	0·070	0·35	0·006	0·94	**−**0·006	0·94
Age (years)	**0·140**	**0·04**	**−**0·001	0·99	**−**0·015	0·84	**−**0·011	0·90
Gender (female, male)	**−**0·027	0·69	0·097	0·23	0·091	0·25	**−**0·070	0·46
Egg intake at T1 (eggs/month)	**0·610**	**<0·01**						
Protein intake (total) at T1 (g/d)			**0·716**	**<0·01**				
Protein intake (animal sources) at T1 (g/d)					**0·718**	**<0·01**		
Adverse effects at T1							**0·408**	**<0·01**
Protein intake (total) at T3 (g/d)	**0·322**	**<0·01**					0·141	0·10
BMI atT3 (kg/m^2^)	0·079	0·23	**−**0·038	0·63	**−**0·029	0·71	0·099	0·28
Physical activity at T3 (kJ)†	0·025	0·71	**−**0·081	0·31	**−0·191**	**0·02**	**−**0·098	0·29
HR QoL score at T3‡	**−**0·070	0·29	**−**0·066	0·40	**−**0·099	0·20	**−0·196**	**0·045**
Previous participant (no, yes)	0·130	0·05	**−**0·002	0·98	0·087	0·27	0·177	0·06

HR QoL, health-related quality of life.

* Significant co-efficients are in bold.

† Physical activity was measured by the Community Healthy Activities Model Program for Seniors questionnaire.

‡ HR QoL was measured by the SF-36 questionnaire.

#### Egg intake

Using the egg consumption FFQ, at T2, group membership (intervention/control) did not predict egg intake (*β* = −0·013, *P* = 0·84). Higher egg intake at T2 was instead associated with higher egg intake at T1, higher protein intake at T2 and a higher BMI at T2 (smallest *β* = 0·154, *P* = 0·02).

Higher egg intake at T3 was significantly associated with being in the intervention group (compared with the control group) (*β* = −0·124, *P* = 0·047). Higher egg intake at T3 was also associated with a higher egg intake at T1, a higher protein intake at T3 and a higher age (smallest *β* = 0·140, *P* = 0·04).

Similar results were also found in egg intake as measured by the SCG-FFQ. These analyses are provided in online Supplementary Material III.

#### Protein intake

Neither total protein intake nor animal protein intake at T2 were predicted by group membership (largest *β* = 0·095, *P* = 0·21). Higher total and animal protein intake at T2 were instead associated with a higher total and animal protein intake at T1, respectively (smallest *β* = 0·681, *P* < 0·01).

Neither total protein intake nor animal protein intake at T3 were predicted by group membership (largest *β* = 0·070, *P* = 0·35). Higher total protein intake at T3 was associated with a higher total protein intake at T1 (*β* = 0·716, *P* < 0·01), and higher animal protein intake at T3 was associated with a higher animal protein intake at T1 and lower physical activity at T3 (smallest *β* = −0·191, *P* = 0·02).

#### Adverse events

Neither adverse events at T2 or T3 were significantly predicted by group membership (largest *β* = 0·051, *P* = 0·56). A higher number of adverse events at T2 were associated with more adverse events at T1 (*β* = 0·409, *P* < 0·01), and a higher number of adverse events at T3 were associated with a higher number of adverse events at T1 and a lower health-related quality of life score (smallest *β* = −0·196, *P* = 0·05).

### Secondary outcomes

No group effects (intervention/control) were found in any of the secondary outcomes (lean body mass, physical performance or muscle strength) at either T2 or T3 (largest *β* = −0·106, *P* = 0·12). Other relationships were as would be expected. Regression analyses for all secondary outcomes are provided in online Supplementary Material IV.

## Discussion

This parallel group, randomised controlled trial investigated the impact of providing high-protein egg-based recipes and single-use herb/spice packets over 12 weeks on egg intake, protein intake, adverse events, lean body mass and several functional measures of lean body mass. Egg intake increased by the end of the 12-week intervention in both groups (intervention/control), and after a further 12 weeks, this higher egg intake was sustained in the intervention group, while egg intake in the control group returned to baseline levels. No other differences between groups were found.

Our total sample was similar to that gained for other studies in community-dwelling older adults^(72,73)^, and randomisation resulted in comparable intervention and control groups. Our target sample size was gained, drop out was low (9 %) and adherence throughout the trial was good.

The findings in egg intake suggest that the provision of high-protein egg-based recipes and single-use herb/spice packets can impact on egg intakes up to 12 weeks after an intervention. Sustained intakes were only found in the intervention group, and these findings suggest that the continued consumption of higher numbers of eggs by this group was a direct result of the intervention. It is very plausible that recipe provision results in sustained behaviour changes; various studies suggest recipe use and reuse over time is possible and likely^(39–44)^. These findings suggest that recipe and single-use herb/spice provision may be particularly useful in the longer term. Follow-up of our participants over the longer term would clearly be of interest. Effect sizes are also meaningful. Our intervention resulted in a 20 % increase in egg intake that was sustained for at least 12 weeks, and this effect was found despite a high egg intake at baseline. A mean baseline egg intake of twenty-two eggs per month is higher than that reported in the National Diet and Nutrition Survey data, where British older adults (65+ years) are reported to consume an equivalent of 16–17 eggs/month^(50)^. Our effects were also found where only 58 % of participants reported using the recipes. Recipes of greater appeal or incentives that result in increased use may have enhanced our findings further.

The findings in adverse events also suggest that our intervention was acceptable and resulted in acceptable changes to diets, where these were achieved. These findings highlight the benefits of a behavioural intervention, where participants can choose to undertake as little or as much of the intervention as they wish (regardless of researcher or practitioner requests). Low rates of adverse events are also likely to have contributed to our sustained effects.

It is unclear, however, whether the recipes and herb/spice packets increased intakes during the intervention period. Some contribution to increased egg intakes during the intervention period may have stemmed from the recipe and herb/spice packet provision, but the similar increase in egg intakes in both groups suggests that equivalent activities in both groups may provide more plausible explanations. Notably, both groups (intervention and control) were part of an intervention study and completed dietary FFQ and questionnaires specifically focusing on eggs as our outcome measures. Uninstructed changes in lifestyles are common in health-related intervention studies, where simply taking part in a study can encourage participants to behave differently^(74,75)^. Both groups also received a dietary information postcard with a short message about the importance of protein for older adults, and eggs were included in this message amongst a number of other protein-rich foods. Nutrition education interventions with older adults, however, are known to demonstrate only limited impacts on behaviour^(76)^, and overall protein intakes did not increase in either group. Finally, all participants undertook the intervention between June and December in the UK, and it is possible that seasonal influences on dietary intake resulted in the increased egg intake in both groups. Sales information can suggest slight (around 10 %) increases in egg sales when the weather is colder, as occurs in the UK towards December (British Egg Industry Council, unpublished results).

While promising effects in egg intakes were found, no differences were found in total protein intake or in protein intake from animal-based foods, either over time or between groups. The absence of effects in protein intake in combination with the effects in egg intake may suggest firstly that the protein provided by the increased egg intake was insufficient to impact on overall protein intakes. An increase in egg intake of 4–5 eggs/month would provide a mean additional 0·8–1 g protein/d which is small compared with baseline intakes of 92 g/d. These levels *may* be meaningful in older adults of much lower protein intakes, such as those at risk of malnutrition, but an intervention of higher impact would clearly be beneficial for these individuals.

Secondly, however, these findings may suggest that the increased egg consumption did not *add* to existing protein intakes, but *replaced* the consumption of other high-protein foods. Our recipes were designed to increase egg and protein intake specifically at breakfast, through the provision of 25–30 g protein. The 25–30 g protein requirement, however, resulted in the inclusion of recipes that many individuals reported were too large for breakfast or another non-main meal. Our recipes were typically composed of two eggs, plus at least two portions of other protein-rich foods, such as ham, cheese or yogurt. Replacement of an existing high-protein meal with an egg-based meal would increase egg consumption, but probably have limited effects on total protein intakes. These findings may suggest that a recommendation to consume 25–30 g protein in three meals/d is unrealistic for many older adults. A number of studies suggest preferences for smaller portion sizes or reduced appetites with age^(31,34,63,77)^.

Third, an absence of effects on protein intake may also be attributed, at least in part, to high protein intakes at baseline. Our baseline daily protein intake of 92 g/d is high compared with 69·8 g/d in the National Diet and Nutrition Survey data^(50)^. These high protein intakes most likely reflect the active and healthy nature of study volunteers who were only eligible for the study if they were able to come to the University^(78)^. Inflation as a result of over-reporting on our FFQ may also have occurred, but the SCG-FFQ has previously been validated for assessing individual food intakes^(52)^.

Fourth, while only egg intake was impacted by our intervention, egg intake, total protein intake and protein intake from animal-based foods both after the intervention and after follow-up were strongly influenced by intake at baseline. These findings demonstrate the stability of dietary intake in older adults, as has repeatedly been demonstrated elsewhere^(31–34)^, and testify to the difficulty of changing dietary patterns among this age group. Given the difficulty of changing dietary behaviour, our intervention that resulted in a sustained 20 % increase in egg intake over 12 weeks is possibly very valuable.

Given the absence of effects in protein intake, the absence of effects in lean body mass or any of our functional measures of lean body mass is unsurprising. These effects were hypothesised assuming increases in protein intake^(1,2,9)^.

### Implications

Overall, the provision of high-protein egg-based recipes and single-use herb/spice packets resulted in a sustained increase in egg consumption following the end of the intervention, but these increases did not translate into increases in protein intake or increases in lean body mass, strength or function. These findings suggest some benefit from a recipe-based intervention, and we suspect the sustained impact of the intervention results from the ongoing nature of an intervention that is implemented by target individuals themselves and can be repeated as often as they wish. Strategies to increase repeated use, such as the use of laminated recipe cards or the inclusion of a binder to hold multiple recipe cards, may further add to this type of intervention.

The impact of an intervention such as this as part of everyday life remains unknown. Our intervention was intended to mimic real life, where recipe cards (and single-use herb/spice packets) are sometimes provided in supermarkets or in magazines. Our intervention differs from some others using recipes in that cooking classes or required foods were not also provided^(79)^, and greater effects may have been found had eggs or more of the required foods been provided. Our intervention also differs from others through our focus on conventional protein-rich foods as opposed to the use of fortified foods or supplements. Greater increases in protein intakes have been found in dietary studies using fortified foods or supplements (e.g. Ref. 7), but these types of foods can be expensive and are not often acceptable to more able older adults^(20)^, hence our decision to use a food-based approach. Effects in studies using supplements or food provision may also be unlikely to continue once a study stops and supplements/foods are no longer provided.

### Strengths and limitations

The strengths of our study include the inclusion of a sample of similar demographic and lifestyle characteristics to other studies in community-dwelling older adults^(72,73)^, randomisation to comparable intervention and control groups based on these characteristics, and high levels of adherence throughout the trial. Our outcomes were assessed using standard assessments, and associations between variables were found where expected, for example, positive correlations were found between egg intake and protein intake, between gender and handgrip strength. Our intervention was also implemented by over half of those provided with the opportunity. Our egg consumption FFQ was more comprehensive than standard measures and so was more likely to detect changes across the intervention than less sensitive measures. Both the food diaries used in the National Diet and Nutrition Survey, and an FFQ based on only three questions without consideration of egg-based dishes, are likely to underestimate egg consumption as a food that is often irregular in consumption and often consumed as a component part of dishes^(31,78)^. If over-reporting on the egg consumption FFQ may have occurred following the completion of multiple questions, any effects are unlikely to have differed systematically between intervention and control groups or over time. Our study may also suffer from social desirability bias, through the use of self-report questionnaires, but again any effects are unlikely to have differed systematically between intervention and control groups or over time.

Our study was limited by the nature of the specific population taking part, where baseline egg and protein intakes were higher than expected. Including only individuals with low egg intakes or low protein intakes at baseline may have been beneficial for demonstrating effects, but the generalisability of the study would then also be reduced. Greater effects may also have been found had participants been asked or told to use the recipes, but the intervention was intended to mimic a possible real-life intervention, thus this would not have been appropriate. It is difficult to generalise our findings to community-dwelling older adults who are less able or to older adults in residential or care settings, but our intervention was not intended for this population. The study was also limited by the lack of assessment of culinary knowledge and culinary skills, and some other factors that may impact on the eating habits of older individuals, such as physical disabilities^(61,63)^, but we have no reason to believe that these characteristics were likely to differ between our two study groups. Specific recipes for specific target groups, for example, ‘easy to prepare’ recipes for older adults with lower culinary skills, may be beneficial. We can also make no suggestion of the relative value of the recipes and the single-use herb/spice packets, either during the intervention period, or after single-use herb/spice packets had been used. Taste and flavour can be important determinants of food intake in older adults^(31–34)^; thus, additional investigation of the discrimination or synergy of the impacts of recipes and the herb/spice packets would be of interest. We also have no measure of egg or protein intake per meal. Our intervention was intended to increase egg and protein intake at breakfast but our use of an FFQ does not allow us to detect intake per meal. Further assessment may have been of interest, but this would have increased the number of measures and possible demand characteristics further. Previous work, furthermore, demonstrates poor compensation for earlier intakes in older adults^(25,80)^; thus, impacts on breakfast intakes are likely to contribute to overall intakes.

## Conclusion

To conclude, the provision of high-protein egg-based recipes and single-use herb/spice packets over 12 weeks was successful in increasing egg intake up to 12 weeks after the end of the intervention. In both intervention and control groups, egg intake increased during the intervention, but this increase was sustained in the intervention group, while egg intake in the control group returned to baseline levels. The lack of effects in adverse events also suggests that the intervention was acceptable. Protein intake, lean body mass, and the functional measures of lean body mass, however, were not impacted, suggesting that egg consumption replaced as opposed to added to existing dietary protein sources. Our findings suggest that providing recipes and herb/spice packets to older adults can influence eating behaviour, thus providing older adults with recipes could be a useful strategy to change eating behaviour on a population-wide basis.

## Supplementary material

For supplementary material accompanying this paper visit https://doi.org/10.1017/S1368980020002712. click here to view supplementary material
