# Supplementary material for: The provision of recipes and single-use herb/spice packets to increase egg and protein intake in community-dwelling older adults: a randomised controlled trial
Source: Public Health Nutr. 2020 Sep 1;24(8):2109–22. doi: 10.1017/S1368980020002712 (PMC8145475; doi:10.1017/S1368980020002712)
Supplement: Supplementary file 1 [file S1368980020002712sup001.docx]

**THE PROVISION OF RECIPES AND SINGLE-USE HERB / SPICE PACKETS TO INCREASE EGG AND PROTEIN INTAKE IN COMMUNITY-DWELLING OLDER ADULTS:** **A RANDOMIZED CONTROLLED TRIAL**

**Supplementary Materials I: List of recipes per week**

| **Week 1** |  | **Week 2** |
| --- | --- | --- |
| Cheese and ham eggy bread |  | Salmon scrambled egg |
| Spinach, feta and pine nut omelette |  | Ham and egg cobbler |
| Egg cupcakes |  | Croque Madame |
| Tuna and broccoli omelette |  | Mushroom and goat's cheese tortilla |
| Fried egg naan with masala beans |  | Buck rarebit |
| Baked eggs with goat's cheese on ciabatta |  | Turkish eggs with Turkish toast |
| **Week 3** |  | **Week 4** |
| Moroccan spiced eggs and tomatoes with a minted yoghurt |  | Turkish Scrambled eggs |
| Pizza omelette |  | Smoked mackerel scramble |
| Salmon and watercress frittata |  | Special dippy eggs and soldiers |
| Leftover roast chicken crust less mini quiches |  | Smoked salmon and asparagus omelette |
| Eggs Florentine |  | Indian omelette |
| Chinese fried eggs |  | Pesto egg and ham Danish pastry |
| **Week 5** |  | **Week 6** |
| Huevos rancheros |  | Masala scrambled eggs |
| Breakfast wrap |  | Australian eggs Benedict |
| Spinach omelette with salmon |  | Cheese and bacon eggy bread bake |
| Chilli cheese jalapeno omelette |  | Avocado and egg quesadilla with salmon |
| Quinoa scrambled eggs |  | Cherry tomato and parmesan frittata |
| Smoked salmon egg pots |  | Soufflé omelette, quark cream and berries |

**Supplementary Materials II: Subscales of SF-36:**

Supplementary table II. Means and standard deviations for SF-36 subscales per group per time point. Measures are reported as Mean ± SD.

|  | T1 | T2 | T3 |
| --- | --- | --- | --- |
| Intervention group (N = 53) |  |  |  |
| Physical functioning | 79 ± 24 | 78 ± 23 | 77 ± 25 |
| Role limitations due to physical health | 69 ± 41 | 77 ± 39 | 78 ± 37 |
| Role limitations due to emotional problems | 94 ± 22 | 94 ± 23 | 90 ± 27 |
| Energy/fatigue | 63 ± 17 | 69 ± 20 | 67 ± 19 |
| Emotional wellbeing | 83 ± 12 | 86 ± 11 | 84 ± 12 |
| Social functioning | 89 ± 20 | 90 ± 17 | 87 ± 19 |
| Pain | 74 ± 24 | 75 ± 23 | 75 ± 25 |
| General health | 72 ± 18 | 73 ± 19 | 70 ± 16 |
| Health change | 57 ± 20 | 56 ± 20 | 53 ± 17 |
| Control group (N = 47) |  |  |  |
| Physical functioning | 84 ± 16 | 86 ± 15 | 84 ± 18 |
| Role limitations due to physical health | 80 ± 34 | 85 ± 29 | 86 ± 29 |
| Role limitations due to emotional problems | 91 ± 23 | 89 ± 28 | 91 ± 24 |
| Energy/fatigue | 64 ± 19 | 64 ± 18 | 64 ± 19 |
| Emotional wellbeing | 81 ± 14 | 83 ± 13 | 83 ± 12 |
| Social functioning | 89 ± 20 | 92 ± 16 | 90 ± 18 |
| Pain | 75 ± 21 | 78 ± 21 | 79 ± 20 |
| General health | 73 ± 17 | 72 ± 17 | 71 ± 19 |
| Health change | 51 ± 15 | 53 ± 17 | 52 ± 17 |

**Supplementary Materials III: Egg intake from the SCG FFQ**

Egg intake data from the egg consumption FFQ were strongly correlated with egg intake from the SCG-FFQ at each time point (smallest r = .773, p < .01). For the intervention group, T1 egg intake measured by the SCG FFQ was 16.20 + 13.81, T2 egg intake was 18.28 + 15.11, and T3 egg intake was 21.00 + 19.45. For the control group, T1 egg intake was 15.41 + 12.06, T2 egg intake was 17.09 + 14.88, and T3 egg intake was 15.67 + 12.58. It should be noted that the egg intake values from the SCG FFQ are lower than the values from the new scale.

Multiple linear regressions using the SCG FFQ egg intake data show egg intake at T2 was predicted by the regression model, R = .829, R^2^ = .687, adjusted R^2^ = .656, F(9, 89) = 21.744, p < .01. Egg intake at T2 was not significantly predicted by the condition (Beta = -.037, p = .54). Higher egg intake at T2 was predicted by higher egg intake at T1 (Beta = .700, p < .01), higher protein intake at T2 (Beta = .193, p < .01), and being a previous participant (Beta = .158, p = .02). SCG FFQ egg intake at T3 was also significantly predicted by the regression model, R = .817, R^2^ = .667, adjusted R^2^ = .633, F(9, 89) = 19.820, p < .01. Higher egg intake at T3 was significantly predicted by being in the intervention group (Beta = -.161, p = .01), higher egg intake at T1 (Beta = .634, p < .01), higher protein intake at T3 (Beta = .276, p < .01), higher BMI at T3 (Beta = .160, p = .02), lower physical activity at T3 (Beta = -.159, p = .02), and being a previous participant (Beta = .151, p = .024). Beta and p values can be found in Supplementary Table III.

Supplementary Table III: Beta and p values

|  | SCG FFQ egg intake at T2 | | SCG FFQ egg intake at T3 | |
| --- | --- | --- | --- | --- |
| Regression model | R = .829, R^2^ = .687, adjusted R^2^ = .656, F(9, 89) = 21.744, p < .01 | | R = .817, R^2^ = .667, adjusted R^2^ = .633, F(9, 89) = 19.820, p < .01 | |
|  | Beta | p | Beta | p |
| Condition (intervention, control) | -.037 | .54 | -.161 | .01 |
| Age (years) | .050 | .44 | .080 | .23 |
| Gender (female, male) | -.028 | .67 | .038 | .58 |
| Egg intake at T1 (eggs/month) | .700 | < .01 | .634 | < .01 |
| Protein intake (total) (g/day) | .193 | < .01 | .276 | < .01 |
| BMI (kg/m2) | .045 | .47 | .160 | .02 |
| Physical activity (kcal) * | -.090 | .17 | -.159 | .02 |
| HR-QoL score ** | .023 | .72 | -.024 | .72 |
| Previous participant (no, yes) | .158 | .02 | .151 | .02 |

**Supplementary Materials IV: Secondary Outcomes (Lean body mass and functional measures of lean body mass):**

**Lean body mass**

Neither lean muscle mass at T2 nor T3 were associated with group membership (intervention / control) (largest Beta = .002, p = .85). A higher lean body mass at T2 was related to a higher lean body mass at T1 (Beta = .970, p < .01). A higher lean body mass at T3 was related to higher lean body mass at T1 (Beta = .948, p < .01). Beta and p values can be found in Supplementary Table IV and V.

**Functional measures of lean body mass**

Neither physical performance (SPPB) scores, leg extensions, nor handgrip strength at T2 or at T3 were predicted by group membership (largest Beta = -.106, p = .12). Higher SPPB scores at T2 were predicted by lower age (Beta = -.187, p = .01), higher SPPB score at T1 (Beta = .650, p < .01), and higher physical activity at T2 (Beta = .223, p < .01). A higher SPPB score at T3 was significantly related to younger age (Beta = -.151, p = .04), higher SPPB score at T1 (Beta = .571, p < .01), lower BMI at T3 (Beta = -.210, p < .01), higher physical activity at T3 (Beta = .255, p < .01), and higher HR QoL at T3 (Beta = .164, p = .02). More leg extensions at T2 and T3 were related to higher numbers of leg extensions at T1 (smallest Beta = .641, p < .01), and leg extensions at T3 were also related to protein intake at T3 (Beta = .161, p = .04), and physical activity at T3 (Beta = .180, p = .03). Higher handgrip strength at T2 and T3 was significantly related to being male (smallest Beta = .145, p = .001), and higher handgrip strength at T1 (smallest Beta = .849, p < .01). Beta and p values can be found in Supplementary Table IV and V.

Supplementary table IV – Multiple linear regression results predicting lean body mass, physical performance, handgrip strength, and leg extensions after the 12 week intervention (T2) (N=100).

|  | Lean body mass | | Physical performance (SPPB) score | | Handgrip strength | | Leg extensions | |
| --- | --- | --- | --- | --- | --- | --- | --- | --- |
| Regression model | R = .994, R^2^ = .988, adjusted R^2^ = .987, F(9, 89) = 817.778, and p < .01 | | R = .831, R^2^ = .691, adjusted R^2^ = .660, F(9, 89) = 22.129, and p < .01 | | R = .968, R^2^ = .937, adjusted R^2^ = .930, F(9, 89) = 146.511, and p < .01 | | R = .763, R^2^ = .583, adjusted R^2^ = .540, F(9, 88) = 13.655, and p < .01 | |
|  | Beta | p | Beta | p | Beta | p | Beta | p |
| Condition (intervention, control) | -.002 | .85 | -.056 | .39 | -.015 | .58 | -.018 | .80 |
| Age (years) | -.016 | .24 | **-.187** | **.01** | -.053 | .07 | -.075 | .32 |
| Gender (female, male) | .024 | .32 | -.025 | .71 | **.145** | **.01** | .047 | .55 |
| Lean body mass at T1 (kg) | **.970** | **<.01** |  |  |  |  |  |  |
| SPPB score at T1 |  |  | **.650** | **<.01** |  |  |  |  |
| Handgrip strength at T1 (kg) |  |  |  |  | **.861** | **<.01** |  |  |
| Leg extensions at T1 |  |  |  |  |  |  | **.722** | **<.01** |
| Protein intake (total) at T2 (g/day) | -.008 | .51 | -.022 | .72 | -.053 | .06 | -.037 | .60 |
| BMI at T2 (kg/m2) | .001 | .93 | -.068 | .28 | .025 | .39 | -.052 | .48 |
| Physical activity at T2 (kcal) * | .003 | .79 | **.223** | **.01** | -.005 | .88 | .099 | .19 |
| HR-QoL score at T2** | .011 | .38 | .125 | .07 | -.032 | .27 | -.072 | .36 |
| Previous participant (no, yes) | .000 | .99 | -.070 | .29 | -.004 | .89 | -.053 | .48 |

*Physical activity was measured by the CHAMPS questionnaire.

**Health related quality of life was measured by the SF-36 questionnaire.

Supplementary table V – Multiple linear regression results predicting lean body mass, physical performance, handgrip strength, and leg extensions after the 12 week follow-up (T3) (N=100).

|  | Lean body mass | | Physical performance (SPPB) score | | Handgrip strength | | Leg extensions | |
| --- | --- | --- | --- | --- | --- | --- | --- | --- |
| Regression model | R = .992, R^2^ = .985, adjusted R^2^ = .983, F(9, 89) = 646.364, and p < .001 | | R = .808, R^2^ = .652, adjusted R^2^ = .617, F(9, 89) = 18.541, and p < .001 | | R = .966, R^2^ = .934, adjusted R^2^ = .927, F(9, 89) = 138.873, and p < .001 | | R = .723, R^2^ = .523, adjusted R^2^ = .474, F(9, 88) = 10.706, and p < .001 | |
|  | Beta | p | Beta | p | Beta | p | Beta | p |
| Condition (intervention, control) | -.002 | .91 | -.106 | .12 | -.040 | .15 | -.026 | .74 |
| Age (years) | -.028 | .07 | **-.151** | **.04** | -.038 | .21 | -.085 | .29 |
| Gender (female, male) | .047 | .08 | -.114 | .10 | **.161** | **<.01** | .009 | .91 |
| Lean body mass at T1 (kg) | **.948** | **<.01** |  |  |  |  |  |  |
| SPPB score at T1 |  |  | **.571** | **<.01** |  |  |  |  |
| Handgrip strength at T1 (kg) |  |  |  |  | **.849** | **<.01** |  |  |
| Leg extensions at T1 |  |  |  |  |  |  | **.641** | **<.01** |
| Protein intake (total) at T3 (g/day) | -.006 | .67 | .076 | .23 | -.037 | .19 | **.161** | **.04** |
| BMI atT3 (kg/m2) | .002 | .89 | **-.210** | **.02** | .021 | .48 | -.080 | .31 |
| Physical activity at T3 (kcal) * | -.001 | .95 | **.255** | **<.01** | -.014 | .65 | **.180** | **.03** |
| HR QoL score at T3** | .019 | .18 | **.164** | **.02** | -.010 | .73 | -.050 | .53 |
| Previous participant (no, yes) | -.007 | .64 | -.012 | .86 | -.010 | .73 | -.074 | .36 |

*Physical activity was measured by the CHAMPS questionnaire.

**Health related quality of life was measured by the SF-36 questionnaire.
